# Coronary computed tomography angiography-based endothelial wall shear stress in normal coronary arteries

**DOI:** 10.1007/s10554-022-02739-0

**Published:** 2022-10-18

**Authors:** Jussi Schultz, Inge J. van den Hoogen, Jurrien H. Kuneman, Michiel A. de Graaf, Vasileios Kamperidis, Alexander Broersen, J. Wouter Jukema, Antonis Sakellarios, Sotirios Nikopoulos, Konstantina Tsarapatsani, Katerina Naka, Lampros Michalis, Dimitrios I. Fotiadis, Teemu Maaniitty, Antti Saraste, Jeroen J. Bax, Juhani Knuuti

**Affiliations:** 1 Turku PET Centre, Turku University Hospital and University of Turku, Kiinamyllynkatu 4-8, 20520 Turku, Finland; 2 Department of Cardiology, Leiden University Medical Center, Leiden, The Netherlands; 3 Department of Cardiology, AHEPA Hospital, Aristotle University of Thessaloniki, Thessaloniki, Greece; 4 Department of Radiology, Division of Image Processing, Leiden University Medical Center, Leiden, The Netherlands; 5 Netherlands Heart Institute, Utrecht, The Netherlands; 6 Department of Biomedical Research, FORTH-IMBB, Ioannina, Greece; 7 Department of Materials Science and Engineering, Unit of Medical Technology and Intelligent Information Systems, University of Ioannina, Ioannina, Greece; 8 Department of Cardiology, Medical School, University of Ioannina, Ioannina, Greece; 9 Heart Center, Turku University Hospital and University of Turku, Turku, Finland

**Keywords:** Atherosclerosis, Coronary artery disease, Endothelial wall shear stress, Computational fluid dynamics, Coronary computed tomography angiography

## Abstract

Endothelial wall shear stress (ESS) is a biomechanical force which plays a role in the formation and evolution of atherosclerotic lesions. The purpose of this study is to evaluate coronary computed tomography angiography (CCTA)-based ESS in coronary arteries without atherosclerosis, and to assess factors affecting ESS values. CCTA images from patients with suspected coronary artery disease were analyzed to identify coronary arteries without atherosclerosis. Minimal and maximal ESS values were calculated for 3-mm segments. Factors potentially affecting ESS values were examined, including sex, lumen diameter and distance from the ostium. Segments were categorized according to lumen diameter tertiles into small (< 2.6 mm), intermediate (2.6–3.2 mm) or large (≥ 3.2 mm) segments. A total of 349 normal vessels from 168 patients (mean age 59 ± 9 years, 39% men) were included. ESS was highest in the left anterior descending artery compared to the left circumflex artery and right coronary artery (minimal ESS 2.3 Pa vs. 1.9 Pa vs. 1.6 Pa, p < 0.001 and maximal ESS 3.7 Pa vs. 3.0 Pa vs. 2.5 Pa, p < 0.001). Men had lower ESS values than women, also after adjusting for lumen diameter (p < 0.001). ESS values were highest in small segments compared to intermediate or large segments (minimal ESS 3.8 Pa vs. 1.7 Pa vs. 1.2 Pa, p < 0.001 and maximal ESS 6.0 Pa vs. 2.6 Pa vs. 2.0 Pa, p < 0.001). A weak to strong correlation was found between ESS and distance from the ostium (ρ = 0.22–0.62, p < 0.001). CCTA-based ESS values increase rapidly and become widely scattered with decreasing lumen diameter. This needs to be taken into account when assessing the added value of ESS beyond lumen diameter in highly stenotic lesions.

## Introduction

While systemic risk factors play a key role in the development of coronary artery disease (CAD), the site-specific emergence of atherosclerotic lesions depends on local hemodynamical parameters [1]. Endothelial wall shear stress (ESS) has been identified as one of the key components in the formation and long-term evolution of atherosclerotic lesions [2, 3]. ESS is defined as the tangential force per unit area exerted on the vessel wall by the blood flow in the artery. Areas of low ESS such as lateral walls of bifurcations are more prone to the development of plaque [4, 5]. In more advanced stages of CAD, lesions subject to low ESS will undergo plaque progression and specifically show an increase in necrotic core [6–9]. High ESS, on the other hand, is associated with adverse cardiac events [7, 10–12]. However, only limited data are available regarding ESS values in coronary arteries without atherosclerosis. Also, most of the previous work has been based on invasive imaging modalities [13, 14], although the feasibility of coronary computed tomography angiography (CCTA)-based ESS when compared to invasive methods has been demonstrated [15]. Therefore, the purpose of our study is to evaluate CCTA-based ESS in coronary arteries without signs of atherosclerosis, and to assess factors affecting ESS values.

## Methods

### Study design and population

Consecutive patients referred for a clinically-indicated CCTA due to suspected CAD at the Turku University Hospital, Turku, Finland between 2007 and 2011 were investigated for this analysis [16]. For the present study, CCTA images of 172 patients were evaluated and coronary arteries without atherosclerotic lesions were identified. ESS calculations for the vessels were independently performed by a separate core laboratory.

### CCTA acquisition

CCTA imaging procedures were reported in detail previously [16, 17]. All computed tomography (CT) scans were performed with a 64-row hybrid positron emission tomography (PET)-CT scanner (GE Discovery VCT or D690, General Electric Medical Systems, Waukesha, Wisconsin). To reach the target heart rate of less than 60 bpm as well as maximal vasodilation, intravenous metoprolol (0–30 mg) and isosorbide dinitrate aerosol (1.25 mg) or sublingual nitrate (800 µg) were administered prior to the scans. Intravenously administered low-osmolar iodine contrast agent (48–155 ml; 320–400 mg/ml) was used. Whenever possible, prospectively triggered acquisition was applied to reduce radiation dose. The images were taken in diastole whenever enabled by a sufficiently low heart rate. The resolution of the images in the x–y direction was approximately 0.4 mm with slight variations due to the patient-specific adjustment of the field-of-view. The slice thickness was 0.625 mm, whereas the image matrix size was 512 × 512.

### Image analysis

CCTA images were first visually inspected by the imaging physician at the Turku University Hospital, Turku, Finland. The absence of atherosclerosis was defined by visual inspection in multiple views of the coronary arteries. The main epicardial arteries, namely, the left anterior descending (LAD), left circumflex artery (LCx) and right coronary artery (RCA) were included. The side branches were not analyzed.

### Computational fluid dynamics and endothelial wall shear stress

**Fig. 1:**
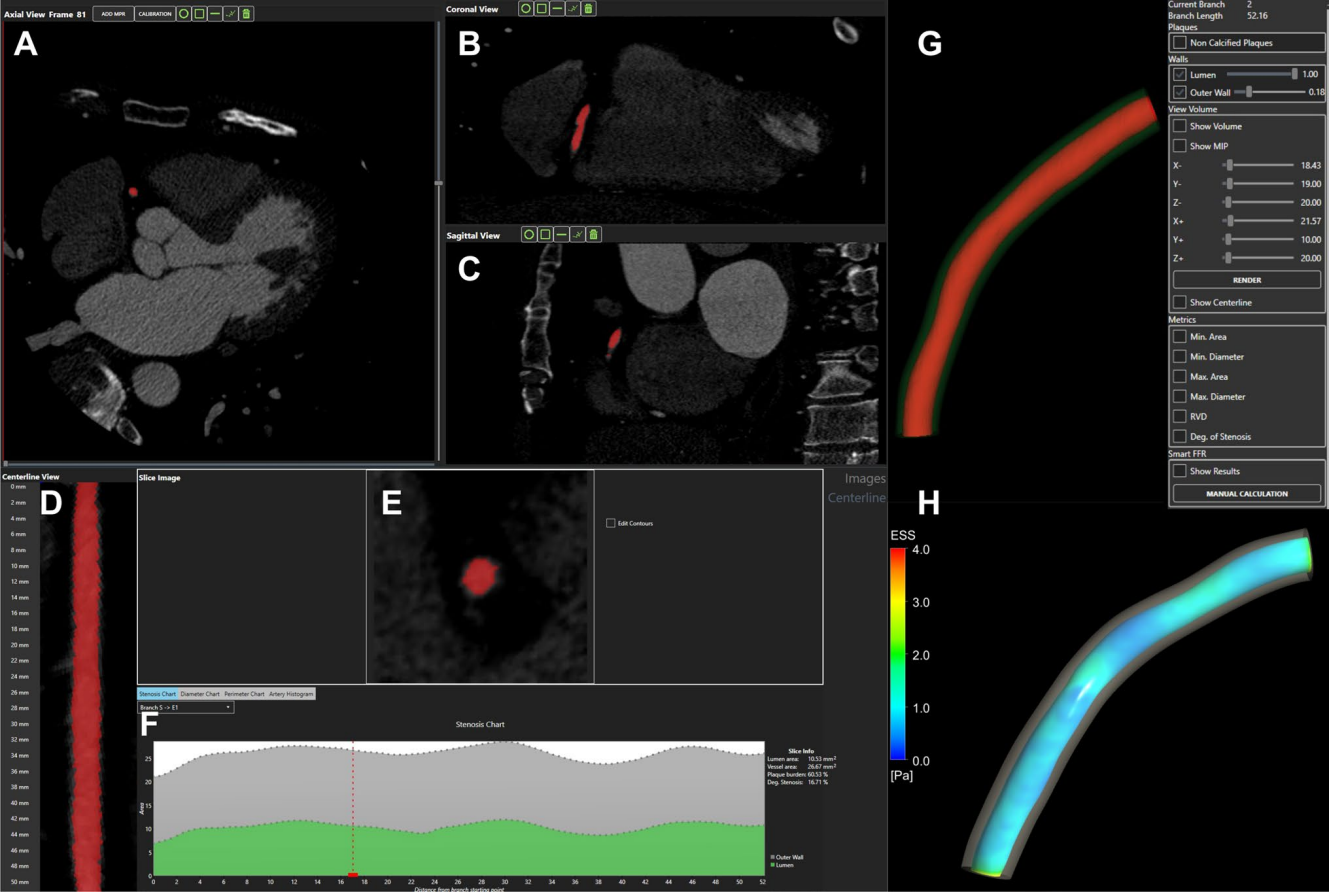
Illustration of the 3D reconstruction and blood flow simulation. **A–C** Axial, coronal and sagittal views of CCTA frame, respectively. **D** Centerline view of the reconstructed segment, **E**: cross section of the lumen area, **F** Stenosis chart depicting the lumen and outer wall areas, **G** The 3D reconstructed lumen and outer wall of the RCA, **H**: ESS distribution

An illustration of the 3D reconstruction and blood flow simulation is given in Fig. 1. An expert cardiologist from the University Hospital of Ioannina, Greece performed the 3D reconstruction of the coronary arteries, using previously developed and validated software for CCTA imaging [18–20]. LAD and LCx were reconstructed after the bifurcation separately and treated independently. The left main artery (LM) was excluded from the simulations. A tetrahedral mesh was then created for each 3D arterial model [21]. Steady state flow simulations based on the incompressible Navier–Stokes equations were performed using finite element commercial software (ANSYS CFX version 18.1, Canonsburg, Philadelphia) [22]. Blood was assumed to be a Newtonian fluid with dynamic viscosity of 0.0035 Pa*s and density of 1050 kg/m^3^, the vessel wall was assumed to be rigid and a no-slip boundary condition was applied [23, 24]. A fixed mean pressure of 100 mmHg was used as the inlet boundary condition, and a uniform velocity profile of 1 ml/s was used as the outlet condition based on previous publications reporting this as the mean flow rate [25–28]. Assessment of ESS was done using dedicated software (SMARTool version 0.9.17, FORTH, Ioannina, Greece) [18]. ESS was calculated as the product of viscosity and the velocity gradient. The values were first calculated for 0.5-mm cross-sections, which were then combined into 3-mm segments. Average values were calculated over 90° arcs, and minimal and maximal ESS were calculated as the minimum and maximum of such averages around the circumference of the vessel. In addition, the mean lumen areas of the 3-mm segments were calculated. Lumen diameters for each segment were calculated from the lumen areas, assuming a circular shape of the vessel cross-sections. The feasibility of the method has been studied previously [18]. In particular, the time required for the 3D reconstruction scaled linearly with the length of the vessel, with a reconstruction time of ~ 1 min for a 90-mm vessel. The required time for the ESS calculations for such a vessel was ~ 20 min.

### Statistical methods

Continuous variables are reported as mean ± standard deviation (SD) or median (interquartile range (IQR)). Categorial variables are reported as counts (percentage). For all statistical analyses, the 3-mm segments were treated as independent observations.

First, factors potentially affecting ESS values were examined: (1) sex, (2) lumen diameter, and (3) distance from the ostium. For LAD and LCx, the distance from ostium was measured from the bifurcation. Segments were categorized according to lumen diameter tertiles into small (< 2.6 mm), intermediate (2.6–3.2 mm) or large (≥ 3.2 mm) segments. The Kruskal–Wallis test was used for three-group comparisons, while pairwise comparisons were performed using the Wilcoxon Rank Sum test with the Bonferroni correction. Also, analysis of covariance (ANCOVA) was used to compare ESS according to epicardial artery and sex, while controlling for the effect of lumen diameter. In the ANCOVA, a logarithmic transformation was performed on lumen diameter, minimal ESS, and maximal ESS to achieve linearity, and all post-hoc analyses were performed using Tukey’s method. The association between ESS and distance from the ostium was evaluated using Spearman’s correlation coefficients.

Second, three linear regression models were built to evaluate the dependence of ESS on the various factors. The first model included the epicardial artery and lumen diameter as explanatory variables [(log(min/max ESS) ~ artery + log(diameter)], the second model included the epicardial artery and distance from the ostium [(log(min/max ESS) ~ artery + distance + artery:distance], and the third model was a combination of the two [(log(min/max ESS) ~ artery + log(diameter) + distance + artery:distance]. The effect of lumen diameter on ESS was assumed to be independent of the epicardial artery in question, and therefore the corresponding interaction term was not included. Comparison of nested models was done using analysis of variance (ANOVA).

Last, normal ranges of minimal and maximal ESS values were calculated per epicardial artery and lumen diameter using the following steps: (1) a logarithmic transformation was performed to achieve normality, (2) a normal distribution was fitted to the data with the normal range computed as mean ± 2SD, and (3) the range was transformed back with an exponential transformation. Vessels with < 10 mm of length analyzed, segments with lumen diameter < 1.5 mm, and the first 3 mm segments of each vessel were excluded to account for artificial values due to entrance length effects and limited image and mesh resolution.

All statistical tests were two-tailed, and p-values < 0.05 were considered statistically significant. P-values for the correlation coefficients were calculated via the asymptotic T approximation. All statistical analyses were performed with R (version 3.6.2, R Development Core Team, Vienna, Austria) [29].

## Results

### Patient and vessel characteristics

**Fig. 2:**
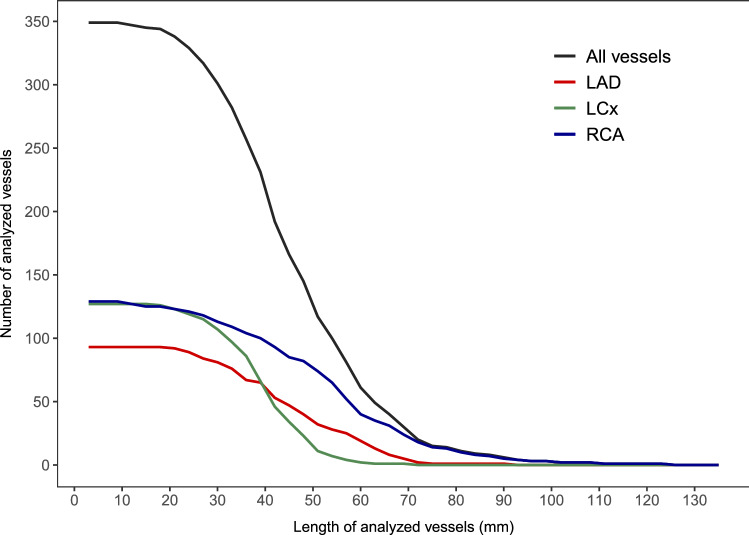
Length of successfully analyzed vessels. The number of vessels which were successfully analyzed beyond a given vessel length. Different graphs shown for all vessels and the main epicardial arteries. *LAD* left anterior descending artery, *LCx* left circumflex artery, *RCA* right coronary artery

In the studied patients, 357 coronary arteries without atherosclerosis were identified, from which 8 vessels were excluded due to unsuccessful ESS analysis (n = 7) or ambiguity regarding normal status (n = 1). Hence, 349 vessels from 168 patients (mean age of 59 ± 9 years, 39% men) were included. Baseline patient characteristics are summarized in Table 1. Of the analyzed vessels, 93 were LAD, 127 LCx and 129 RCA Table 2. This resulted in a total of 5223 analyzed 3-mm segments. Figure 2 illustrates the feasibility of ESS calculation in terms of the length of the successfully analyzed vessels. The overall median length of the analyzed vessels was 42 mm (IQR 33–54 mm). Analysis of the RCA was the longest (54 mm, IQR 39–63 mm), followed by the LAD (45 mm, IQR 33–57 mm) and LCx (39 mm, IQR 33–45 mm).

**Table 1:** Baseline patient characteristics of study population

Characteristic	n = 168
Age, years	59 ± 9
Male	65 (39)
BMI, kg/m^2^	26.2 (24.3–29.1)
Symptoms	
Typical angina	38 (23)
Atypical angina or non-cardiac pain	122 (73)
Dyspnea at exertion	51 (30)
Cardiac risk factors	
Hypertension	81 (48)
Dyslipidemia	100 (60)
Diabetes mellitus	16 (10)
Family history of CAD	78 (46)
Smoking history	49 (29)
Cardiac medication	
Anti-platelet drug	88 (52)
Beta blockers	79 (47)
Calcium channel blocker	20 (12)
Renin-angiotensin system inhibitors	47 (28)
Statins	67 (40)
Laboratory findings	
Total cholesterol, mmol/l	5.0 (4.3–5.6)
Low-density lipoprotein, mmol/l	2.7 (2.1–3.3)
High-density lipoprotein, mmol/l	1.6 (1.3–1.9)
Triglycerides, mmol/l	1.2 (0.9–1.6)
Creatinine, µmol/l	74 (65–85)

Mean ± SD, median (IQR) or n (%) are reported. *BMI* body mass index, *CAD* coronary artery disease

**Table 2:** Vessel characteristics of study population

Characteristic	n = 349
Number of analyzed vessels	
LAD	93 (27)
LCx	127 (36)
RCA	129 (37)
Length of analyzed vessels, mm	
All	42 (33–54)
LAD	45 (33–57)
LCx	39 (33–45)
RCA	54 (39–63)

Median (IQR) or n (%) are reported. *LAD* left anterior descending artery, *LCx* left circumflex artery, *RCA* right coronary artery

### Endothelial wall shear stress

The overall median values of minimal and maximal ESS over the analyzed vessels were 1.8 Pa (IQR 1.2–3.0 Pa) and 2.8 Pa (IQR 2.0–4.9 Pa), respectively. The overall median lumen diameter was 2.9 mm (IQR 2.5–3.3 mm). Regarding the epicardial arteries, the LAD had the highest values of both minimal and maximal ESS (2.3 Pa and 3.7 Pa, respectively), compared to the LCx (1.9 Pa and 3.0 Pa) and RCA (1.6 Pa and 2.5 Pa) Table 3. All pairwise comparisons were statistically significant (p < 0.001). The RCA had the highest median lumen diameter of 3.1 mm, with the LAD and LCx having medians of 2.75 mm and 2.81 mm, respectively (p = 0.015 for LAD vs. LCx, p < 0.001 for others). After adjusting for lumen diameter in the ANCOVA model, statistically significant pairwise differences in minimal and maximal ESS were observed between the epicardial arteries (p < 0.001).

**Table 3:** Endothelial wall shear stress and lumen diameter (n = 5223 segments)

	All n = 5223	LAD n = 1390	LCx n = 1604	RCA n = 2229	p-value*
Minimal ESS, Pa	1.8 (1.2–3.0)	2.3 (1.4–4.3)	1.9 (1.3–3.4)	1.6 (1.1–2.3)	< 0.001
Maximal ESS, Pa	2.8 (2.0–4.9)	3.7 (2.3–7.1)	3.0 (2.0–5.7)	2.5 (1.8–3.6)	< 0.001
Lumen diameter, mm	2.9 (2.5–3.3)	2.8 (2.3–3.2)	2.8 (2.4–3.2)	3.1 (2.6–3.4)	< 0.001

^*^Kruskal–Wallis test

Medians (IQR) are reported. *ESS* endothelial wall shear stress, *LAD* left anterior descending artery, *LCx* left circumflex artery, *RCA* right coronary artery

### Factors affecting endothelial wall shear stress

#### ESS versus sex

Men had lower values for both minimal and maximal ESS compared to women (minimal ESS 1.7 Pa vs. 1.9 Pa, p < 0.001 and maximal ESS 2.7 Pa vs. 2.9 Pa, p = 0.044) Table 4. Conversely, the median lumen diameter was larger in men compared to women (3.1 mm vs. 2.8 mm, p < 0.001). The differences in ESS remained statistically significant after adjusting for lumen diameter in the ANCOVA model (p < 0.001).

**Table 4:** Factors affecting endothelial wall shear stress (n = 5223 segments)

	Sex			Lumen diameter			
	Male n = 2063	Female n = 3160	p-value*	Small (< 2.6 mm) n = 1741	Intermediate (2.6–3.2 mm) n = 1741	Large (≥ 3.2 mm) n = 1741	p-value*
Minimal ESS, Pa	1.7 (1.2–2.8)	1.9 (1.3–3.2)	< 0.001	3.8 (2.4–6.6)	1.7 (1.3–2.3)	1.2 (0.9–1.6)	< 0.001
Maximal ESS, Pa	2.7 (1.9–4.6)	2.9 (2.0–5.1)	0.044	6.0 (3.8–10.1)	2.6 (2.0–3.6)	2.0 (1–6-2.6)	< 0.001
Lumen diameter, mm	3.1 (2.6–3.5)	2.8 (2.4–3.2)	< 0.001	–	–	–	–

^*^Kruskal–Wallis test. Medians (IQR) are reported

*ESS* endothelial wall shear stress, *LAD*, left anterior descending artery, *LCx* left circumflex artery, *RCA* right coronary artery

#### ESS versus lumen diameter

**Fig. 3:**
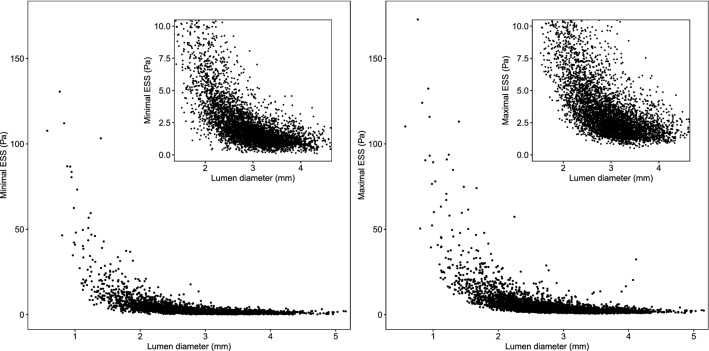
Relationship between ESS and lumen diameter. Minimal ESS (left) and maximal ESS (right) as functions of lumen diameter. *ESS* endothelial wall shear stress

The relationship between ESS and the lumen diameter is illustrated in Fig. 3, and the ESS values according to lumen diameter tertiles are presented in Table 4. Small segments clearly stood out with 3.8 Pa (IQR 2.4–6.6 Pa) for minimal ESS and 6.0 Pa (IQR 3.8–10.1 Pa) for maximal ESS. For the intermediate segments, minimal and maximal ESS were 1.7 Pa (IQR 1.3–2.3 Pa) and 2.6 Pa (IQR 2.0–3.6 Pa), respectively, whereas the large segments had the lowest and least scattered values with 1.2 Pa (IQR 0.9–1.6 Pa) for minimal ESS and 2.0 Pa (IQR 1.6–2.6 Pa) for maximal ESS. The differences in ESS between size classes were all statistically significant (p < 0.001).

#### ESS versus distance from the ostium

**Fig. 4:**
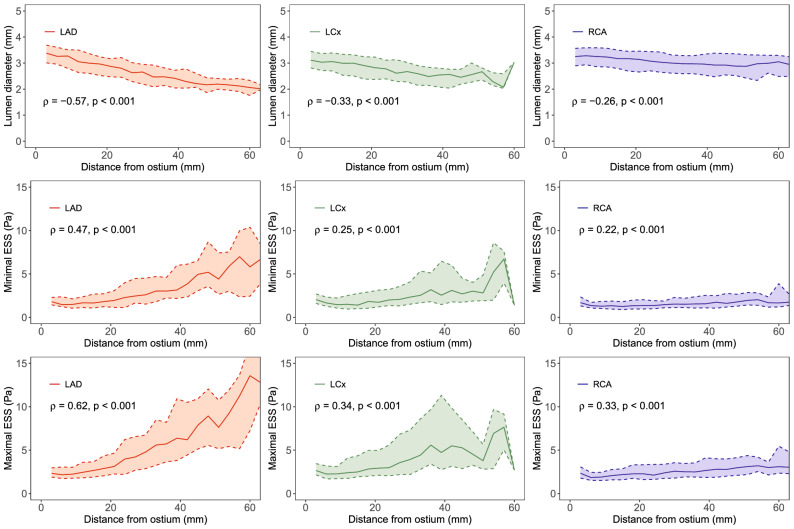
Relationship between ESS and distance from the ostium. Medians (solid line) and interquartile ranges (shaded area) of lumen diameter (top), minimal ESS (middle) and maximal ESS (bottom) as functions of distance from the ostium for the LAD (red), LCx (green) and RCA (blue). Spearman’s correlation coefficients (ρ) are also reported. *ESS* endothelial wall shear stress, *LAD* left anterior descending artery, *LCx* left circumflex artery, *RCA* right coronary artery

The relationship between ESS and distance from the coronary artery ostium is illustrated in Fig. 4. For the LAD, there was a moderate positive correlation between minimal ESS and distance from the ostium (ρ = 0.47, p < 0.001) and a strong positive correlation for maximal ESS (ρ = 0.62, p < 0.001). For the other epicardial arteries, the correlations were considerably weaker with ρ values ranging from 0.22 to 0.34 (p < 0.001). The evaluation of minimal and maximal ESS in the LCx was unreliable after 40 mm due to a small number of analyzed vessels beyond that point.

### Regression models

The first regression model with the epicardial artery and lumen diameter as explanatory variables yielded R^2^ values of 0.612 for minimal ESS (p < 0.001) and 0.611 for maximal ESS, (p < 0.001). The second regression model with the epicardial artery and distance from the ostium was able to explain less of the variance in ESS (R^2^ = 0.150 for minimal ESS, p < 0.001, and R^2^ = 0.240 for maximal ESS, p < 0.001). The addition of the extra variable in the third model resulted in a statistically significant improvement compared to both models (R^2^ = 0.614 for minimal ESS, p < 0.001, and R^2^ = 0.635 for maximal ESS, p < 0.001) (p < 0.001 for both nested model comparisons).

### Range of normal values

The normal ranges of minimal and maximal ESS values are reported in Table 5 per epicardial artery and lumen diameter. The ranges for maximal ESS were consistently wider than those for minimal ESS. As can be clearly seen also in Fig. 3, the ranges became larger when moving to smaller vessel segments. The RCA had the smallest ranges for all vessel sizes, while the widest ranges were observed in the LAD.

**Table 5:** Ranges of minimal and maximal ESS per epicardial artery and lumen diameter

	Small (< 2.6 mm)	Intermediate (2.6–3.2 mm)	Large (≥ 3.2 mm)
Minimal ESS, Pa			
LAD	1.1–17.6	0.6–5.4	0.4–3.6
LCx	1.0–15.3	0.7–4.5	0.4–3.0
RCA	0.9–10.3	0.6–4.0	0.4–2.7
Maximal ESS, Pa			
LAD	2.0–27.7	1.1–8.8	0.8–5.2
LCx	1.6–24.0	1.1–7.6	0.9–4.5
RCA	1.6–13.6	1.1–5.6	0.8–4.7

Vessels with < 1 cm of length analyzed, segments with lumen diameter < 1.5 mm, and the first 3 mm segments of each vessel were excluded from the analyses. *ESS* endothelial wall shear stress, *LAD* left anterior descending artery, *LCx* left circumflex artery, *RCA* right coronary artery

## Discussion

ESS has been identified as one of the key components in the formation and long-term evolution of atherosclerotic lesions [2, 3]. The methodology to measure ESS in human coronary arteries has been developed, but most measurements have been performed invasively. By using noninvasive modalities such as CCTA, one can non-invasively assess the entire coronary artery tree. Some recent studies have analyzed ESS from CCTA images, however, very little is known concerning the values of ESS in normal coronary arteries. Nevertheless, this information is crucial to understand the ESS findings in atherosclerotic coronary arteries.

In the present study, we evaluated ESS in 349 coronary arteries without atherosclerosis using CCTA to gain understanding of the behavior of ESS in normal coronary arteries. Although cardiovascular risk factors were present in many patients, and many patients had also atherosclerotic lesions in other vessels (not included in the analysis), our study population represented patients for which information regarding ESS values is the most relevant. Indeed, CCTA is seldom performed on completely healthy individuals.

The ESS values showed a rapid increase along with a decreasing lumen diameter. This was expected based on the fluid mechanics, as the velocity of the blood flow in the artery becomes higher when lumen becomes narrower. In addition, the spread of the distribution became wider in small segments. Our findings showed that ESS distributions varied between the epicardial arteries, as well as slightly between men and women. The differences between different epicardial arteries could be partially explained by the differences in vessel size, as the LAD and LCx taper more rapidly in diameter while the RCA remains fairly constant until very distally in the vessel [30]. However, we have shown that this explanation is not exhaustive. Also, the small differences in men and women were mostly explained by different sizes of the vessels, but not completely.

In earlier literature Doriot et al. analyzed the coronary artery bifurcations of 21 patients undergoing cardiac catheterization [13]. The range of the resulting ESS values was 0.33–1.24 Pa with a mean value of 0.68 Pa. In a more extensive analysis, Soulis et al. reported the detailed topography of ESS in a model of a normal left coronary artery tree from invasive angiography [14]. They observed higher ESS values in the distal LAD as compared to proximal parts of the vessel. This finding was also verified by our analysis. However, it should be noted that this behavior was most clearly seen specifically in the LAD, and for instance in the RCA, the dependence of ESS on the location along the vessel was less obvious. This was at least partially due to the more constant lumen diameter of the RCA.

Concerning CCTA-based assessment of ESS, Hetterich et al. studied the ESS distributions of 7 patients with non-obstructive CAD (< 30% diameter stenosis) [31] 67.9% of cross-sections in 10 successfully analyzed vessels were non-diseased, corresponding to a mean ESS value of 1.66 ± 0.84 Pa (range 0.02–13.75 Pa). As can be seen from Fig. 4, the range of ESS values obtained in our study was considerably wider. This may be due to intrinsic differences in the used algorithms but could also be the result of different location and size of the analyzed vessels.

A study comparing ESS values obtained from CCTA to those from invasive coronary angiography (ICA) was done by Huang et al. [15]. They studied 41 patients with mild or moderate coronary stenosis who underwent both CCTA and ICA, and found good correlation between the ESS values derived from the two modalities. In fact, the mean ESS values were 4.97 Pa (4.37–5.57 Pa) vs. 4.86 Pa (4.27–5.44 Pa), the minimal ESS values were 0.86 Pa (0.67–1.05 Pa) vs. 0.79 Pa (0.63–0.95 Pa), and the maximal ESS values were 14.50 Pa (12.62–16.38 Pa) vs. 13.76 Pa (11.44–16.08 Pa). All differences were statistically nonsignificant. These results are therefore in line with our reported values for small lumen diameters.

## Limitations

Our findings were part of an observational study with inherent limitations. Our study does not provide direct comparison of CCTA based ESS values with those obtained using invasive methdos. The measurements in our study were typically done during diastole, and therefore the change in lumen diameter could not be taken into account. We also used a constant velocity for all vessels in our simulations, since the use of CCTA imaging instead of invasive methods resulted in the lack of actual velocity data. It is known, that ESS depends not only on the lumen diameter, but also on the specific 3D geometry of the vessel [14]. Our analysis did take the curvature of vessels into account, but the effect of bifurcations was not investigated in detail. The lack of side branches indicates that the simulated flow in the distal parts of the vessels is higher than in reality, and this can result in unrealistically high ESS values as seen in our study. However, if ESS would be used for predictive purposes, the differences in the predictive accuracy of disease progression is minor compared to ESS assessed at arteries which include the side branches [32]. Finally, the ESS analysis process was not feasible in all vessels, which was especially the case in distal segments likely due to limited resolution of CCTA and motion artefacts.

## Conclusions

We derived ESS values for visually normal coronary arteries from CCTA images. CCTA-based ESS values increase rapidly and become widely scattered with decreasing lumen diameter. This needs to be taken into account when assessing the added value of ESS beyond lumen diameter in highly stenotic lesions. Further studies are needed to determine which factors need to be accounted for when studying ESS in stenotic lesions.

## Abbreviations

ANCOVA: Analysis of covariance
ANOVA: Analysis of variance
BMI: Body mass index
CAD: Coronary artery disease
CCTA: Coronary computed tomography angiography
CT: Computed tomography
ESS: Endothelial wall shear stress
ICA: Invasive coronary angiography
IQR: Interquartile range
LAD: Left anterior descending artery
LCx: Left circumflex artery
LM: Left main artery
PET: Positron emission tomography
RCA: Right coronary artery
SD: Standard deviation
